# The association between urinary nitrate and prostate specific antigen without prostatic disease: a cohort study

**DOI:** 10.1186/s12894-025-01878-5

**Published:** 2025-08-09

**Authors:** Zhiyi Chen, Enpu Zhang, Lu Gan, Wenjuan Zhang, Guixiao Huang

**Affiliations:** Department of Urology, The Third Affiliated Hospital of Shenzhen University, Shenzhen Luohu Hospital Group Luohu People’s Hospital, Shenzhen, 518000 China

**Keywords:** PSA, Urinary nitrate, PCa, NHANES

## Abstract

**Purpose:**

The carcinogenic effects of nitrate reduction to nitrite are well established, and nitrate has been closely linked to various diseases. However, research on its effects on the urinary system remains limited, and its association with PSA remains unclear. This study aims to investigate the relationship between urinary nitrate and PSA.

**Methods:**

We employed weighted generalized linear models, weighted univariate analysis, forest plots, weighted multivariable analysis, and generalized additive models (GAM) to investigate the relationship between urinary nitrate and PSA. Subgroup analyses were conducted to further validate the stability of this association across different groups. GAM was utilized to provide a more intuitive representation of the specific relationships between PSA and urinary nitrate.

**Results:**

TPSA, FPSA, and the prostate specific antigen ratio (%, 4 ng/mL ≤ TPSA ≤ 10 ng/mL) showed negative correlations with urinary nitrate. After adjusting for confounders, only the prostate-specific antigen ratio maintained a significant negative association (β = -632.7; 95% CI: -1094.9, -170.6; *P* = 0.011), while correlations with TPSA and FPSA were not statistically significant. A nonlinear relationship was observed, where urinary nitrate initially remained stable or changed minimally before gradually declining.

**Conclusion:**

This study identifies a negative correlation between urinary nitrate and the prostate-specific antigen ratio in individuals with gray-zone TPSA. These findings enhance our understanding of urinary nitrate’s impact and may help reduce overtreatment in this population.

## Article Highlights

1. This study is the first to investigate the relationship between urinary nitrate and the prostate-specific antigen (PSA) ratio in a general population without prostatic disease, providing novel insights into the potential impact of urinary nitrate on PSA screening.
2. Our findings reveal a significant negative correlation between urinary nitrate and the PSA ratio in the gray zone (4–10 ng/mL), suggesting that urinary nitrate may improve the accuracy of prostate cancer screening and reduce overtreatment.
3. The study highlights the importance of exploring urinary biomarkers in prostate cancer screening, potentially leading to more precise risk assessment and fewer unnecessary biopsies.

## Introduction

Prostate cancer ranks as the second leading cause of cancer-related mortality among men and is the most commonly diagnosed malignancy in the male population [1]. Many patients with prostate cancer (PCa) remain asymptomatic and unaware of their condition [2]. PSA testing serves as an initial screening tool for PCa, facilitating early diagnosis and timely intervention [2]. However, numerous studies have indicated that PSA levels may be influenced by various factors [3–5], potentially leading to detection bias. Overdiagnosis or missed diagnoses due to these influencing factors may result in inappropriate and unnecessary treatments [6, 7]. Several studies have demonstrated that repeated PSA testing prior to prostate biopsy, particularly when a PSA reduction of 20% or more is observed, is associated with a lower likelihood of prostate cancer, especially high-grade disease [8]. In efforts to enhance PCa detection while minimizing unnecessary biopsies, researchers have proposed integrating imaging modalities with various biomarkers, such as the Prostate Health Index (PHI), 4Kscore, MyProstateScore (MPS), SelectMDx, ExoDx Prostate IntelliScore (EPI), and IsoPSA [9]. These strategies aim to strike a balance between reducing overdiagnosis and accurately identifying clinically significant cancers. PSA should be taken with caution as detection PCa method of screening [9].

The gold standard for PCa diagnosis is prostate biopsy, an invasive procedure that can cause both physical and psychological distress. This procedure remains particularly controversial for men with PSA levels in the gray zone [10]. When serum total PSA (tPSA) levels fall within the gray zone (4–10 ng/mL), its specificity is relatively low [11] The free-to-total PSA (f/t PSA) ratio is also subject to various pre-analytical and clinical factors that may affect its reliability [12, 13]. Studies have shown that among patients with tPSA levels in the gray zone, the difference in tPSA levels between PCa and non-PCa groups is not significant [14, 15]. Moreover, the concentration of free PSA (fPSA) in cancer patients is often lower than that in benign prostatic hyperplasia (BPH) cases [16]. The f/t PSA ratio has been suggested as a useful marker for distinguishing early-stage PCa from BPH; however, some studies have reported no significant differences in f/t PSA ratios between PCa and non-PCa groups [12]. These findings suggest that f/t PSA in the gray zone is also influenced by other factors. Therefore, identifying risk factors that affect PSA-related markers is of critical clinical importance for improving the accuracy of PCa screening.

Nitrate has been associated with adverse health outcomes, including cancer [17]. It is naturally present in water and soil, and humans primarily ingest it through drinking water and various dietary sources [18]. Ingested nitrate is classified as a probable human carcinogen due to endogenous nitrosation [18]. This process involves the conversion of nitrate to nitrite in the gastrointestinal tract, leading to the synthesis of N-nitroso compounds (NOCs). The intake of antioxidant vitamins and the use of nonsteroidal anti-inflammatory drugs (NSAIDs) inhibit endogenous nitrosation, whereas meat consumption and inflammatory gastrointestinal diseases promote it [19]. Some meta-analyses have highlighted a positive association between dietary nitrate intake (excluding nitrite) and the risk of colorectal [20] and ovarian [21] cancers.

Since urinary nitrate is also present in the urinary system, its potential role in prostate cancer remains largely unexplored. To date, few studies have investigated the relationship between urinary nitrate and PSA. Therefore, this study aims to further explore this association.

## Methods

### Study design and population

The National Health and Nutrition Examination Survey (NHANES) is a comprehensive health and nutrition survey conducted by the National Center for Health Statistics in the United States. Since its initiation in the early 1960s, NHANES has aimed to assess the health and nutritional status of individuals nationwide. Through household interviews and physical examinations, the survey collects extensive data encompassing biological, social, psychological, behavioral, and demographic factors, all of which are freely available to participants. In this cross-sectional study, we utilized NHANES data from 2001 to 2010 to investigate the association between urinary nitrate and PSA in the general population.

### Sample selection

We conducted analyses using the NHANES database, which includes a total of 69,469 participants from 2001 to 2010. The dataset encompasses various demographic variables, including age, race, ratio of family income to poverty, marital status, and education level. Additionally, we considered factors such as C-reactive protein, BMI, serum cotinine, hypertension, high cholesterol level, diabetes, drinking, urinary creatinine, urinary nitrate, and PSA (TPSA, FPSA, and the prostate-specific antigen ratio).

**Fig. 1:**
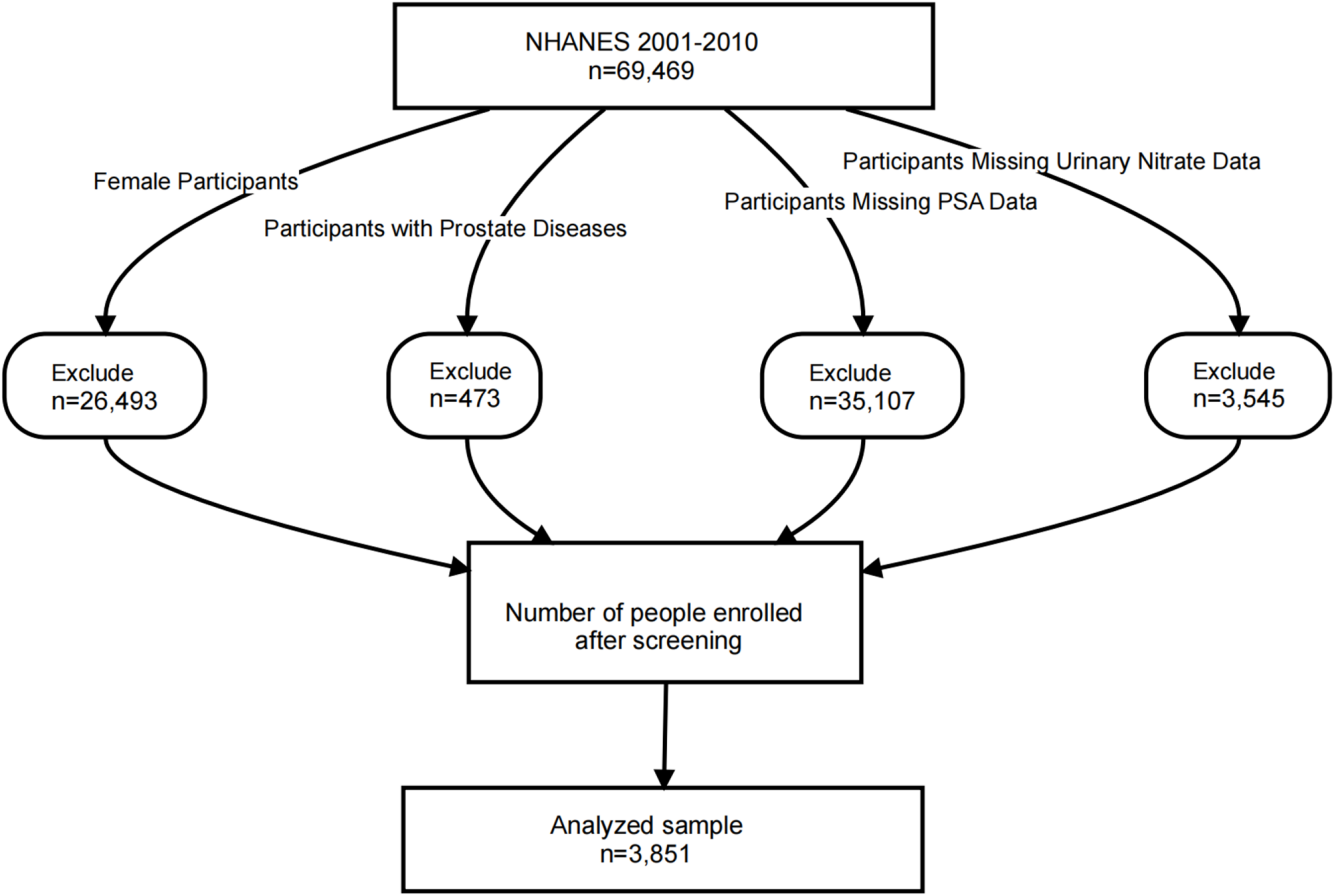
Flow chart of the study population. *NHANES* the National Health and Nutrition Examination Survey

From the initial 69,469 participants, we excluded females (*n* = 26,493), participants with prostate diseases (*n* = 473, including BPH, prostate infections or inflammation, and self-reported malignancies such as prostate cancer), participants with missing PSA data (*n* = 35,107), and those with missing urinary nitrate data (*n* = 3545). Consequently, a total of 3851 participants were included in this cross-sectional study (Fig. 1).

It is noteworthy that all NHANES participants from 2001 to 2010 provided informed consent, and the study protocol was approved by the Research Ethics Review Board of the National Center for Health Statistics.

### Variables

The demographic variables considered in this study were age, race (including Mexican American, other Hispanic, non-Hispanic white, non-Hispanic black, or other race), poverty income ratio (PIR), marital status, and education level. To classify the education level variable, individuals with educational attainment below the 9th grade, those with 9th-11th grade education (including 12th grade without a diploma), and high school graduates or equivalent were categorized as the “High School Grad and Less Than” group. On the other hand, individuals with some college or associate degrees, as well as college graduates or above, were classified as the “Above” group. Thus, the education level variable was dichotomized as “High School Grad and Less Than” versus “Above. Poverty income ratio (PIR) [poverty: PIR ≤ 1, beyond the poverty threshold: PIR > 1].

The questionnaire variables in this study included several factors, such as hypertension, high cholesterol level, diabetes, and drinking. Alcohol use was defined based on individuals who reported consuming at least 12 alcoholic drinks in the past 12 months [22]. Accordingly, the drinking variable was categorized as a binary variable: “Drinking” (“No” or “Yes”), using the 12-drink threshold as the classification criterion.

Laboratory measurements included C-reactive protein (CRP), serum cotinine levels, urinary creatinine, PSA, and urinary nitrate. Smoking status was determined based on serum cotinine levels, with non-smokers defined as having serum cotinine ≤ 10 ng/mL and smokers as having serum cotinine > 10 ng/mL [23]. PSA measurements included total PSA (TPSA), free PSA (FPSA), and the prostate-specific antigen ratio. TPSA, initially treated as a continuous variable, was further categorized into three groups: <4 µg/L, ≥ 4 µg/L & ≤10 µg/L, and > 10 µg/L.

Anthropometric measurements included body mass index (BMI), which was analyzed as a continuous variable. For further analysis, BMI was classified into three categories: <25 kg/m_2_, ≥ 25 kg/m_2_ & <30 kg/m_2_, and ≥ 30 kg/m_2_.

Regarding laboratory measurements, PSA (including TPSA, FPSA, and the prostate specific antigen ratio) and urinary nitrate were analyzed. This prostate specific antigen ratio was calculated by dividing the free PSA by the total PSA: Prostate specific antigen ratio = round ((FPSA/TPSA)*100) (whole number).

For the NHANES laboratory methodology regarding the determination of PSA, further information can be accessed at: https://wwwn.cdc.gov/Nchs/Data/Nhanes/Public/2009/DataFiles/PSA_F.htm (Date of access online: January 19, 2025). Similarly, detailed information regarding the NHANES laboratory methodology for urinary nitrate determination is available at: https://wwwn.cdc.gov/Nchs/Data/Nhanes/Public/2009/DataFiles/PERNT_F.htm (Date of access online: January 19, 2025). Additional covariates can be found at the following URL: https://wwwn.cdc.gov/nchs/nhanes/default.aspx (Date of access online: January 19, 2025).

### Data analysis

Given the intricate probability cluster design of NHANES, individual sample weights were assigned to each survey participant, and all statistical analyses in this study took into account these weights. We conducted statistical analyses in accordance with the guidelines outlined by the Centers for Disease Control (CDC) [14, 24].

All statistical analyses were carried out using Empowerstats (https://www.empowerstats.net/cn/) and R software. All estimates were weighted using appropriate NHANES sample weights. Following guidelines from the Centers for Disease Control and Prevention, weighted models were used to address the oversampling of minority ethnicities, ensuring fair and accurate estimates of the population impact. Results were considered statistically significant when p-values were less than 0.05.

## Results

Table 1 presents 17 variables, including the independent variables TPSA, FPSA, and prostate specific antigen ratio (%); the dependent variable urinary nitrate (µg/L); and relevant covariates. The covariates include age, C-reactive protein, urinary creatinine, education level, race, marital status, ratio of family income to poverty, BMI, hypertension, high cholesterol level, diabetes, drinking status, and serum cotinine. TPSA was categorized into three groups: <4 ng/mL, ≥ 4 ng/mL to ≤ 10 ng/mL, and > 10 ng/mL, with the corresponding mean ages of 54.6, 67.3, and 69.1 years, respectively. For continuous variables, weighted means (95% CI) were calculated, and P-values were determined using survey-weighted linear regression (svyglm). For categorical variables, weighted percentages (95% CI) were computed, and P-values were obtained using survey-weighted chi-square tests (svytable).

**Table 1:** Characteristics of participants, weighted (*N* = 3581)

TPSA (ng/mL)	< 4	≥ 4, ≤ 10	> 10	*P*-value
Urinary nitrate (ug/L)	59248.2 (56612.0,61884.4)	53656.2 (48047.0,59265.4)	46181.4 (36342.3,56020.6)	0.009
TPSA (ng/mL)	1.1 (1.0,1.1)	5.7 (5.5,6.0)	17.6 (15.5,19.8)	< 0.001
FPSA (ng/mL)	0.3 (0.3,0.3)	1.2 (1.1,1.3)	2.6 (2.0,3.2)	< 0.001
Prostate specific antigen ratio (%)	31.5 (30.8,32.1)	21.0 (19.9,22.1)	17.0 (12.7,21.3)	< 0.001
Age (years)	54.6 (54.0,55.2)	67.3 (65.9,68.6)	69.1 (64.9,73.2)	< 0.001
C-reactive protein (mg/dL)	0.4 (0.3,0.4)	0.4 (0.3,0.5)	0.6 (0.4,0.8)	0.049
urinary creatinine (mg/dL)	134.2 (130.3,138.0)	133.1 (121.5,144.7)	109.5 (95.6,123.5)	0.004
Education level				0.639
High School Grad and Less Than	44.0 (41.0,47.1)	45.2 (38.0,52.7)	52.0 (31.4,71.9)	
Above	56.0 (52.9,59.0)	54.8 (47.3,62.0)	48.0 (28.1,68.6)	
Marital status				0.561
Married or with partners	76.2 (73.9,78.3)	74.7 (67.0,81.2)	71.6 (57.1,82.7)	
Widowed or divorced	14.6 (12.7,16.6)	17.0 (12.2,23.1)	23.2 (13.0,37.8)	
Unmarried	6.7 (5.6,7.9)	5.2 (2.8,9.4)	3.3 (1.0,10.4)	
Separated	2.6 (1.9,3.5)	3.1 (1.2,8.0)	1.9 (0.5,6.8)	
Ratio of family income to poverty				0.146
≤ 1	9.2 (8.0,10.6)	7.3 (5.1,10.4)	16.3 (6.7,34.6)	
> 1	90.8 (89.4,92.0)	92.7 (89.6,94.9)	83.7 (65.4,93.3)	
BMI (kg/m2)				0.030
< 25	21.3 (19.6,23.1)	26.5 (21.8,31.9)	31.1 (18.8,46.9)	
≥ 25, < 30	42.7 (40.2,45.2)	46.1 (39.2,53.1)	34.1 (23.0,47.3)	
≥ 30	36.0 (33.5,38.5)	27.4 (21.6,34.1)	34.8 (20.1,53.0)	
Hypertension				< 0.001
No	62.7 (60.2,65.0)	46.6 (39.0,54.5)	51.4 (35.0,67.5)	
Yes	37.3 (35.0,39.8)	53.4 (45.5,61.0)	48.6 (32.5,65.0)	
High cholesterol level				0.959
No	50.7 (47.9,53.5)	49.7 (42.6,56.9)	51.7 (34.0,69.0)	
Yes	49.3 (46.5,52.1)	50.3 (43.1,57.4)	48.3 (31.0,66.0)	
Diabetes				0.908
No	88.8 (87.5,90.0)	87.8 (82.6,91.6)	88.3 (68.1,96.4)	
Yes	11.2 (10.0,12.5)	12.2 (8.4,17.4)	11.7 (3.6,31.9)	
Drinking				0.827
No	95.7 (94.3,96.7)	96.2 (90.9,98.5)	97.5 (87.0,99.6)	
Yes	4.3 (3.3,5.7)	3.8 (1.5,9.1)	2.5 (0.4,13.0)	
Serum cotinine(ng/mL)				< 0.001
≤ 10	69.4 (66.6,72.1)	86.2 (81.4,89.9)	87.3 (71.2,95.0)	
> 10	30.6 (27.9,33.4)	13.8 (10.1,18.6)	12.7 (5.0,28.8)	
Race				0.303
Mexican American	6.0 (4.8,7.6)	3.3 (1.9,5.8)	7.1 (3.3,14.6)	
Other Hispanic	3.5 (2.5,4.8)	2.4 (1.3,4.3)	7.3 (1.5,29.0)	
Non-Hispanic White	76.4 (72.7,79.7)	78.8 (72.4,84.1)	67.2 (50.3,80.5)	
Non-Hispanic Black	8.9 (7.3,10.8)	9.8 (6.9,13.8)	14.3 (7.1,26.8)	
Other Race	5.2 (4.0,6.7)	5.7 (2.9,11.0)	4.1 (0.7,21.5)	

Data in the table: For continuous variables: survey-weighted mean (95% CI), P-value was by survey-weighted linear regression (svyglm), For categorical variables: survey-weighted percentage (95% CI), P-value was by survey-weighted Chi-square test (svytable)

Table 2 presents the results of the univariate analysis for 16 variables associated with urinary nitrate. The analysis indicates that serum TPSA, prostate-specific antigen ratio (%, 4 ≤ TPSA ≤ 10), and FPSA are all negatively correlated with urinary nitrate. Additionally, age, smoking status, urinary creatinine, race (Other Race), education level (Above), hypertension, and diabetes also show associations with urinary nitrate levels. Regarding age, urinary nitrate decreases by 662.9 µg/L for each additional year. Among smokers, urinary nitrate levels are 7,520.7 µg/L higher compared to non-smokers. For urinary creatinine, each 1 mg/dL increase corresponds to a 307.4 µg/L rise in urinary nitrate. In terms of race, individuals classified as Other Race exhibit 17,824.9 µg/L higher urinary nitrate levels compared to Mexican Americans. Regarding education, participants with education levels above high school demonstrate an increase of 4,496.7 µg/L in urinary nitrate compared to those with a high school education or lower. Furthermore, individuals with hypertension show a reduction of 32.0 µg/L in urinary nitrate levels, while those with diabetes exhibit a decrease of 10,375.3 µg/L.

**Table 2:** Univariate analysis for urinary nitrate, weighted

Covariate	β(95%CI)	*P*-value
TPSA (ng/mL)	-906.1(-1398.7, -413.6)	< 0.001
TPSA (ng/mL) Tertile		
<4	Ref	
≥ 4, ≤ 10	-5592.0(-11902.9, 718.9)	0.090
>10	-13066.8(-22931.3, -3202.2)	0.012
FPSA (ng/mL)	-7326.0 (-123.1, -4384.6)	< 0.001
Prostate specific antigen ratio (%)	-19.8(-10267.5, 83.5)	0.709
Prostate specific antigen ratio (%, 4ng/mL < = TPSA < = 10ng/mL)	-638.7 (-1072.5, -204.8)	0.006
Age (years)	-662.9(-822.5, -503.4)	< 0.001
C-reactive protein (mg/dL)	408.0(-1734.9, 2551.0)	0.710
Serum cotinine(ng/mL)		
≤ 10	Ref	
> 10	7520.7(2998.3, 12043.2)	0.002
Urinary creatinine (mg/dL)	307.4 (281.1,333.6)	< 0.001
BMI (kg/m2)		
< 25	Ref	
≥ 25, < 30	-2373.4 (-7034.8,2288.1)	0.322
≥ 30	-340.8 (-5189.7,4508.1)	0.891
Marital status		
Married or with partners	Ref	
Widowed or divorced	-1823.4 (-5670.2, 2023.4)	0.357
Unmarried	6429.6 (-2194.6, 15053.9)	0.149
Separated	-5060.8 (-20514.9, 10393.4)	0.523
Ratio of family income to poverty		
≤ 1	Ref	
> 1	-742.6 (-6769.9,5284.7)	0.810
Race		
Mexican American	Ref	
Other Hispanic	3729.4 (-8093.5, 15552.4)	0.539
Non-Hispanic White	-3692.5 (-9294.6, 1909.5)	0.202
Non-Hispanic Black	-4851.1 (-11198.4, 1496.2)	0.140
Other Race	17824.9 (1177.0, 34472.9)	0.040
Education level		
High School Grad and Less Than	Ref	
Above	4496.7 (596.3, 8397.1)	0.027
Hypertension		
No	Ref	
Yes	-32.0 (-49.63, -14.41)	< 0.001
High cholesterol level		
No	Ref	
Yes	-1608.5(-5250.7, 2033.8)	0.390
Diabetes		
No	Ref	
Yes	-10375.3 (-15754.4, -4996.1)	< 0.001
Drinking		
No	Ref	
Yes	2960.3(-7763.1, 13683.6)	0.590

### Subgroup analysis

**Fig. 2:**
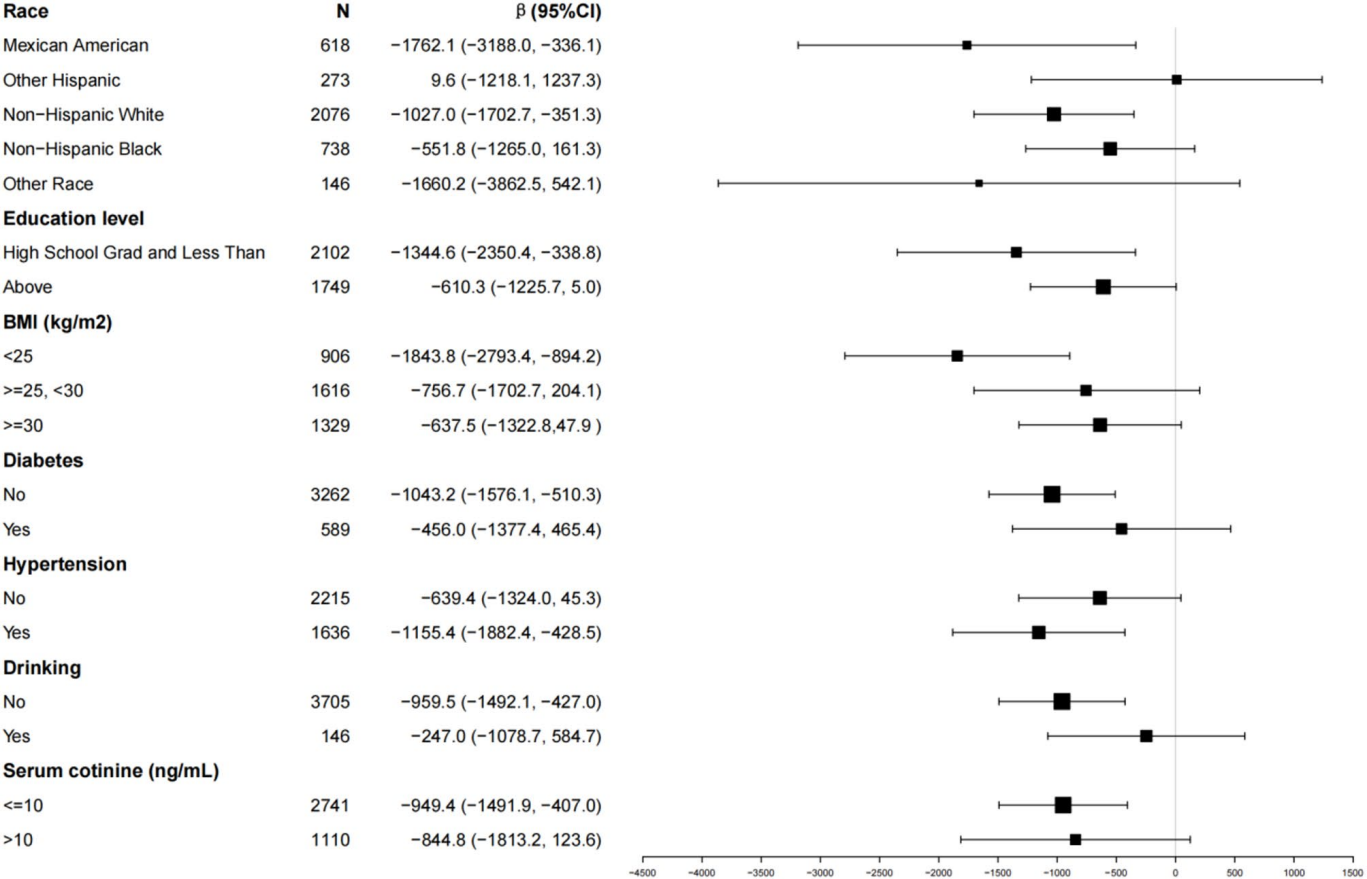
Effects on the correlation between TPSA and urinary nitrate in different subgroups

To further investigate the influence of additional covariates on the association between TPSA and urinary nitrate, Fig. 2 presents the results of a subgroup analysis. Across all subgroups, TPSA remains negatively correlated with urinary nitrate, with the effect being more pronounced in the following subgroups:

Race: Mexican American (β = -1762.1, 95% CI: -3188.0 to -336.1); Non-Hispanic White (β = -1027.0, 95% CI: -1702.7 to -351.3).

Education level: High School Graduate and Less Than (β = -1344.6, 95% CI: -2350.4 to -338.8).

BMI: <25 kg/m_2_ (β = -1843.8, 95% CI: -2793.4 to -894.2).

Diabetes: No (β = -1043.2, 95% CI: -1576.1 to -510.3).

Hypertension: Yes (β = -1155.4, 95% CI: -1882.4 to -428.5).

Drinking: No (β = -959.5, 95% CI: -1492.1 to -427.0).

Serum cotinine: ≤10 ng/mL (β = -949.4, 95% CI: -1491.9 to -407.0).

These findings suggest that the negative correlation between TPSA and urinary nitrate is more substantial in specific demographic and health-related subgroups.

**Fig. 3:**
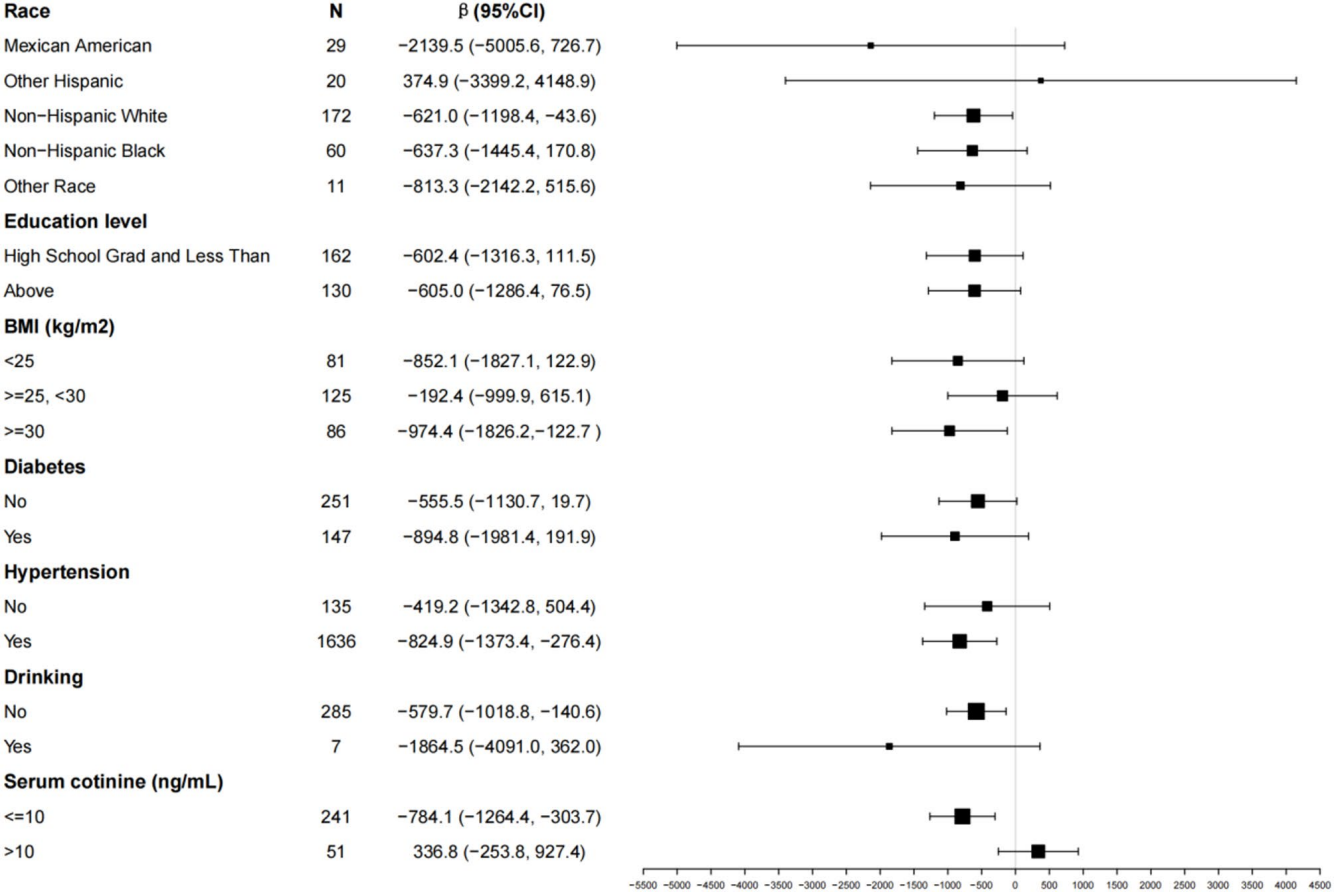
Effects on the association between prostate-specific antigen ratio (%, 4 ng/mL ≤ TPSA ≤ 10 ng/mL)and urinary nitrate in different subgroups

Using Fig. 3, we examined the influence of additional covariates on the association between the Prostate Specific Antigen Ratio (%, 4ng/mL ≤ TPSA ≤ 10ng/mL) and urinary nitrate. In most subgroups, the Prostate Specific Antigen Ratio (%, 4ng/mL ≤ TPSA ≤ 10ng/mL) exhibited a negative correlation with urinary nitrate, with the effect being more pronounced in the following subgroups:

Race: Non-Hispanic White (β = -621.0, 95% CI: -1198.4 to -43.6).

BMI: ≥30 kg/m_2_ (β = -974.4, 95% CI: -1826.2 to -122.7).

Hypertension: Yes (β = -824.9, 95% CI: -1373.4 to -276.4).

Drinking: No (β = -579.7, 95% CI: -1018.8 to -140.6).

Serum cotinine: ≤10 ng/mL (β = -784.1, 95% CI: -1264.4 to -303.7).

These results indicate that the negative association between the Prostate Specific Antigen Ratio and urinary nitrate is more pronounced in specific demographic and health-related subgroups.

Table 3 presents the results of various models. The findings indicate that in the unadjusted model, TPSA, FPSA, and the Prostate Specific Antigen Ratio (%, 4ng/mL ≤ TPSA ≤ 10ng/mL) were all negatively correlated with urinary nitrate, with statistical significance. In the adjusted model, all three variables remained negatively associated with urinary nitrate; however, only the Prostate Specific Antigen Ratio (%, 4 ≤ TPSA ≤ 10) maintained statistical significance (β = -632.7; 95% CI: -1094.9 to -170.6; *P* = 0.011). Specifically, for individuals with TPSA levels between 4 ng/mL and 10 ng/mL, each 1% increase in the Prostate Specific Antigen Ratio was associated with a 632.7 µg/L decrease in urinary nitrate.

**Table 3:** Relationship between PSA (TPSA,FPSA and prostate specific antigen ratio ) and urinary nitrate, weighted

Outcome	Crude Model			Adjusted Model	
	β(95%CI)	*P*-value		β(95%CI)	*P*-value
TPSA (ng/mL)	-906.1 (-1398.7, -413.6)	< 0.001		-256.9 (-635.4, 121.5)	0.191
FPSA (ng/mL)	-7326.0 (-10267.4, -4384.6)	< 0.001		-2393.8 (-4902.3, 114.8)	0.070
Prostate specific antigen ratio (%, 4ng/mL < = TPSA < = 10ng/mL)	-638.7 (-1072.5, -204.8)	0.006		-632.7 (-1094.9, -170.6)	0.011

Data in the table: β(95%CI); P-value

Result variable: Urinary nitrate (ug/L)

Exposure variable: TPSA (ng/mL); FPSA (ng/mL); Prostate specific antigen ratio (%, 4 < = TPSA < = 10)

The adjusted model adjusts for Age; Race (Mexican American, Other Hispanic, Non-Hispanic White, Non-Hispanic Black, Other Race); Education level (High School Grad and Less Than and Above); BMI (≥ 25 kg/m_2_,<30 kg/m_2_ and ≥ 30 kg/m_2_); CRP; Diabetes (No, Yes); Hypertension(No, Yes); Serum cotinine(≤ 10ng/mL,>10ng/mL); Urinary creatinine (mg/dL); Drinking (No, Yes)

**Figs. 4:**
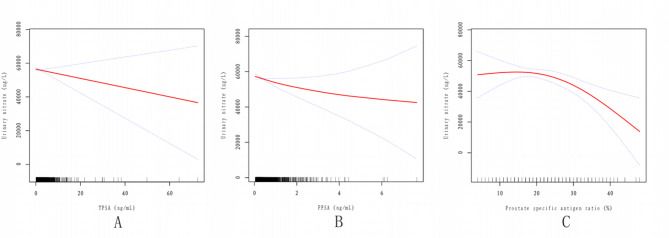
Association between PSA and Urinary nitrate. The Solid red line represents the smooth curve fit between variables. Blue bands represent the 95% confidence interval from the fit. **A** A linear association between TPSA and Urinary nitrate was found in a generalized additive model (GAM). The results were obtained after adjusting for Age; Race (Mexican American, Other Hispanic, Non-Hispanic White, Non-Hispanic Black, Other Race); Education level (High School Grad and Less Than and Above); BMI (≥ 25 kg/m_2_,<30 kg/m_2_ and ≥ 30 kg/m_2_); CRP; Diabetes (No, Yes); Hypertension(No, Yes); Serum cotinine(≤ 10ng/mL,>10ng/mL); Urinary creatinine (mg/dL); Drinking (No, Yes). It can be seen that after adjusting the relevant variables, TPSA was linearly correlated with Urinary nitrate, and Urinary nitrate decreased with the increase of TPSA. **B** The results were obtained after adjusting for Age; Race (Mexican American, Other Hispanic, Non-Hispanic White, Non-Hispanic Black, Other Race); Education level (High School Grad and Less Than and Above); BMI (≥ 25 kg/m_2_,<30 kg/m_2_ and ≥ 30 kg/m_2_); CRP; Diabetes (No, Yes); Hypertension(No, Yes); Serum cotinine (≤ 10ng/mL,>10ng/mL); Urinary creatinine (mg/dL); Drinking (No, Yes). FPSA and urinary nitrate exhibit a negative but nonlinear correlation, wherein urinary nitrate levels decrease with increasing FPSA; however, the rate of decline gradually diminishes. **C** Association between Prostate specific antigen ratio (%, 4ng/mL ≤ TPSA ≤ 10ng/mL) and Urinary nitrate

The results were obtained after adjusting for Age; Race (Mexican American, Other Hispanic, Non-Hispanic White, Non-Hispanic Black, Other Race); Education level (High School Grad and Less Than and Above); BMI (≥ 25 kg/m_2_,<30 kg/m_2_ and ≥ 30 kg/m_2_); CRP; Diabetes (No, Yes); Hypertension(No, Yes); Serum cotinine (≤ 10ng/mL,>10ng/mL); Urinary creatinine (mg/dL); Drinking (No, Yes).

This indicates a nonlinear correlation between the prostate-specific antigen ratio and urinary nitrate. Initially, urinary nitrate levels remain relatively stable or exhibit minimal change as the prostate-specific antigen ratio increases. However, as the ratio continues to rise, urinary nitrate levels gradually decrease, with the rate of decline accelerating over time.

## Discussion

To the best of our knowledge, this is the first nationally representative study investigating the relationship between urinary nitrate and PSA in the general U.S. population without prostate-related diseases (e.g., prostate cancer, benign prostatic hyperplasia, and prostatitis). In the baseline characteristics analysis, TPSA was stratified into three groups (< 4 ng/mL, ≥ 4 ng/mL to ≤ 10 ng/mL, and > 10 ng/mL), revealing statistically significant differences (*P* < 0.05) in TPSA, FPSA, prostate-specific antigen ratio (%), age, C-reactive protein, urinary creatinine, BMI, hypertension, and serum cotinine. Univariate analysis demonstrated a negative correlation between urinary nitrate and TPSA, prostate-specific antigen ratio within the gray zone (4 ng/mL ≤ TPSA ≤ 10 ng/mL), and FPSA. Additionally, age, smoking, urinary creatinine, race (Other Race), education level (Above), hypertension, and diabetes were associated with urinary nitrate levels. After adjusting for covariates and conducting subgroup analyses, a robust independent negative correlation between prostate-specific antigen ratio within the gray zone and urinary nitrate was confirmed. This association was more pronounced in Non-Hispanic White individuals, those with BMI ≥ 30 kg/m_2_, individuals with hypertension, non-drinkers, and non-smokers. Even after adjusting for relevant covariates, the relationship between prostate-specific antigen ratio within the gray zone and urinary nitrate remained stable and statistically significant. Further application of a generalized additive model (GAM) with smooth curve fitting using a two-segment linear regression model revealed that as the prostate-specific antigen ratio within the gray zone increased, the rate of decline in urinary nitrate levels gradually accelerated (Fig. 4-C). Although TPSA and FPSA did not reach statistical significance (*P* > 0.05) in the multivariable regression model, the smooth curve fitting plots indicated a negative linear trend between TPSA, FPSA, and urinary nitrate (Fig. 4-A and -B).

Extensive research has been conducted on the carcinogenic effects of nitrates, though relatively few studies have examined their impact on the urinary system or their potential association with prostate cancer. Some meta-analyses have reported a positive correlation between dietary nitrate exposure (excluding nitrite) and the risk of colorectal and ovarian cancer [21]. Additionally, other meta-analyses have identified a significant association between nitrite intake and an increased risk of gastric cancer [25]. The NIH-AARP Diet and Health Study found a positive correlation between nitrites in processed meats and postmenopausal breast cancer risk [26]. Research by Sinha et al. indicated a positive association between nitrate exposure and prostate cancer [27]. In the Iowa Women’s Health Study, dietary nitrite intake was linked to an elevated risk of bladder cancer [28]. Furthermore, in the same cohort, higher nitrate intake from drinking water and higher nitrite consumption from processed meats were associated with an increased risk of kidney cancer in elderly women [29]. Findings from a Dutch cohort study suggested that higher total nitrite intake was related to an increased incidence of esophageal squamous cell carcinoma [30], while greater nitrite consumption from processed meats was positively associated with pancreatic cancer risk [31]. Moreover, in the male population of the NIH-AARP cohort, higher dietary nitrate intake (but not nitrite intake) was linked to an increased risk of thyroid cancer [32].

In some regions, PSA [2] is a commonly used initial screening biomarker for prostate cancer. Additionally, the microbiota (chronic infection, viral gene incorporation, and microbial metabolites) may also play an important role in the diagnosis, development, and treatment of prostate cancer [33]. Current diagnostic methods for PCa are invasive and lack specificity for aggressive forms of the disease, which can lead to overtreatment. A new class of non-invasive alternatives is being developed, especially the appropriate combination of some biomarkers into multiplexed biosensor platforms to achieve accurate diagnostic tests [34]. However, the shortcomings are obvious and have not yet been addressed in differentiating between aggressive and indolent cancers [34]. PSA density may also play a role in the PSA/PCa debate [35]. Jeffrey J Tosoian and other scholars analyzed the data of 422 patients in the main cohort and 268 patients in the validation cohort and found that the MyProstateScore (MPS) test can effectively evaluate the necessity of repeat biopsy in men with previous negative biopsies. When MPS ≤ 15, it can 100% exclude GG ≥ 2 cancer and avoid 23% unnecessary biopsies. When MPS > 40, it can avoid 67% of biopsies and maintain a negative predictive value of 95%. Although it will delay the diagnosis of GG ≥ 2 cancer by 32%, it can accurately identify high-risk patients [36]. PCa gene 3 (PCA3) is one of these molecular biomarkers, and current evidence shows significant promise in improving some of the limitations of PSA in specific populations. Urine PCA3 testing has shown improved diagnostic performance compared with serum PSA and DRE [37]. Increased BMI has a certain predictive effect on complications after radical surgery for PCa [38]. By identifying these influences, we seek to reduce unnecessary examinations and interventions, particularly for patients within the PSA gray zone. Various factors can contribute to elevated PSA levels, including urinary tract infections and benign prostatic hyperplasia [39]. Given the limited research directly examining the relationship between PSA and urinary nitrate, we hypothesize that potential interactions between these two variables may affect PSA levels. Understanding this relationship could help mitigate confounding effects or interferences in prostate cancer screening, thereby improving the accuracy of risk assessment.

In our study, we systematically excluded individuals with potential confounding conditions affecting PSA levels, such as prostatitis, prostate infections, benign prostatic hyperplasia, and a prior history of prostate cancer. The use of a sufficiently large sample size ensured high statistical power. While this study offers significant advantages, it is crucial to acknowledge and address its limitations. First, the cross-sectional design of the NHANES database restricts our ability to establish causality between serum PSA and urinary nitrate. Second, this study did not consider potential unmeasured confounding variables that could affect PSA and urinary nitrate levels, and our study was not a clinical trial. Perhaps its main limitation is that it is only a retrospective analysis of NHANES. Finally, NHANES did not report urine culture results due to database limitations, which is a limitation of this study. It is essential to consider these limitations when interpreting the findings and to encourage further research to explore these associations in greater depth.

## Conclusion

Our study enhances the understanding of the impact of urinary nitrate exposure on the PSA ratio, particularly within the PSA gray zone. The findings suggest that urinary nitrate primarily affects the prostate specific antigen ratio in this gray zone, potentially aiding in the reduction of overtreatment in this patient population. This research provides a novel perspective on the relationship between urinary nitrate and the prostate specific antigen ratio while emphasizing the importance of further exploration and application of urinary biomarkers in the PSA gray zone. Future studies should validate the quality and potential of urine nitrate in combination with other urine biomarkers to improve prostate cancer screening to increase diagnostic accuracy and reduce unnecessary biopsies.

## Data Availability

The datasets generated during and/or analyzed during the current study are available in the NHANES repository, https://wwwn.cdc.gov/nchs/nhanes/Default.aspx.
